# Construction and Performance Study of BDDE-Toughened Modified Mannich Base Epoxy System

**DOI:** 10.3390/ma19071332

**Published:** 2026-03-27

**Authors:** Siyu Wu, Suining Zheng, Wenlan Zhang, Huaxin Chen

**Affiliations:** 1 School of Materials Science and Engineering, Chang’an University, Xi’an 710061, China; 2020031005@chd.edu.cn (S.W.); zhengsn@chd.edu.cn (S.Z.); 2 Shaanxi Transportation Planning and Design Research Institute Co., Ltd., Xi’an 710065, China; zwl2020030@163.com

**Keywords:** road engineering, anti-skid surface layer, epoxy resin, chain extension toughening, application research

## Abstract

To mitigate the issue of brittleness and cracking in epoxy resin (EP) anti-skid systems, this study investigates four key aspects tailored to application scenarios: toughening, low shrinkage, strong adhesion, and rapid curing at ambient temperature. 1,4-Butanediol diglycidyl ether (BDDE) was used to extend the chain of triethylenetetramine (TETA), followed by a Mannich reaction with formaldehyde (F) and cardanol to prepare a flexible aliphatic amine Mannich base curing agent containing flexible segments (Curing Agent B). The influence of composition ratios on the mechanical properties of the cured product was studied. The curing performance of the epoxy system under various temperature conditions and its adhesion to asphalt substrates were characterized. The thermal shrinkage behavior of the epoxy system under temperature-variable environments was also investigated. The results indicated that the elongation at break of the epoxy curing system, after chain extension and toughening, increased from 28.7% to 40.4%, representing a 28.9% increase. When *n* (Cardanol):*n* (TETA):*n* (F):*n* (BDDE) = 1:1.4:0.8:0.7 (molar ratio of reactants), *m* (EP):*m* (Curing Agent B) = 1:1 (mass ratio), and epoxy-terminated polyurethane (EPU) prepolymer constituted 10% of the epoxy resin mass; the epoxy curing system exhibited an elongation at break of 44.3%, a tensile strength of 7.0 MPa, a bond strength of 6.9 MPa, and an impact toughness of 1.77 J/cm^2^. Furthermore, it exhibited rapid curing at a low temperature (0~5 °C) and at room temperature (25 °C). Additionally, when bisphenol F epoxy resin was used, the system demonstrated optimal thermal expansion properties.

## 1. Introduction

**Figure 1:**
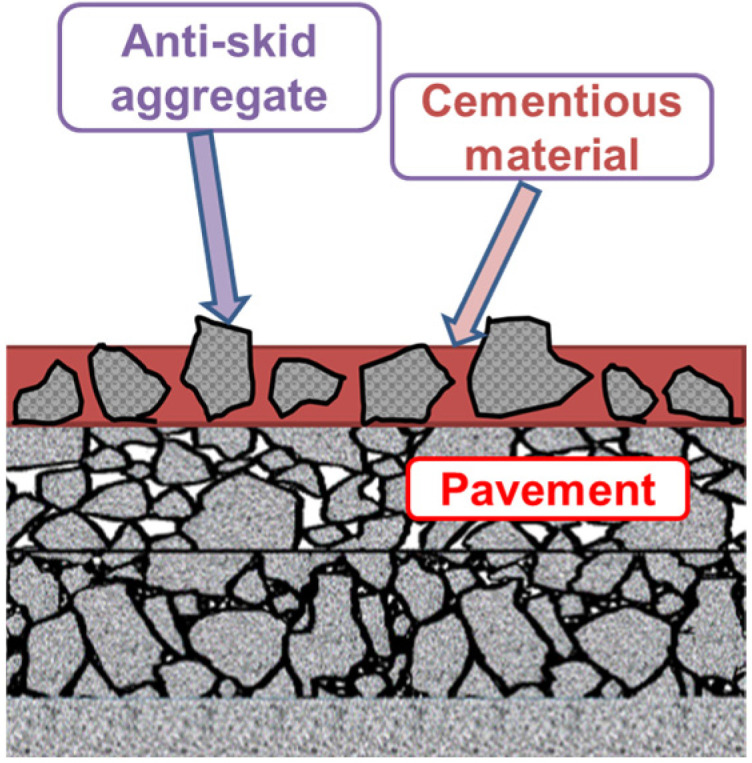
Schematic diagram of anti-skid wearing course.

In recent years, traffic accidents caused by insufficient pavement skid resistance have been frequent, especially in tunnels, curves, long slopes, and bridge decks, which pose a great threat to people’s lives. Therefore, it is particularly important to improve and maintain the skid resistance of existing pavements [1]. Research shows that compared with anti-skid wearing course and slurry seal technology, the anti-skid wearing course technology has the advantages of simple and quick construction and excellent skid resistance and is currently the main technology used to improve pavement skid resistance. The conventional anti-skid wearing course mostly uses EP as the adhesive system, which is evenly applied on the pavement to be maintained, and then a layer of wear-resistant anti-skid aggregate is sprinkled to form a thin layer with anti-skid function [2] (Figure 1).

The commonly used high molecular resin adhesive for anti-skid wearing courses mainly includes epoxy resin, polyurethane and acrylic resin. Among them, epoxy resin occupies a dominant position due to its good storage stability, high-cost performance and wide range of component ratios. However, the brittle and rigid room temperature rapid curing-type epoxy resin anti-skid system has a thermal expansion coefficient significantly higher than that of asphalt pavement, which is prone to cracking after paving and has poor durability. Conversely, the epoxy system with good flexibility has a slow curing speed of the adhesive, resulting in a long traffic closure time after laying [3]. Therefore, it is urgent to study a high-toughness epoxy curing system that can rapidly cure at room temperature. This paper first developed a high-toughness epoxy–adhesive system that can rapidly cure and then selected bauxite clinker with rich resources and excellent anti-skid performance as the anti-skid aggregate to construct a high anti-skid toughening surface treatment system.

The current research on epoxy resin-based anti-skid surfaces shows a notable methodological imbalance: significant advances have been made in compositional optimization—including resin–hardener stoichiometry adjustment, incorporation of polyurethane or rubber particles for toughening [4], dispersion of nano-SiO_2_ for enhanced modulus and reduced shrinkage [5,6], and refinement of aggregate gradation to improve macro-texture [7]. Critical interfacial challenges have been highlighted in recent studies: pull-out tests after fire exposure show that heating to 300 °C reduces epoxy–concrete bond strength by 57.3%, with failure shifting from cohesive to purely interfacial, indicating irreversible degradation of chemical bonds and inter-diffusion zones [8]. Similarly, mismatch in the coefficients of thermal expansion (CTE) among epoxy binder, bauxite clinker aggregate, and concrete substrate leads to persistent interfacial shear stresses during cooling; finite-element modeling confirms that these stresses can reach up to 3.7 times the fracture toughness of the epoxy–aggregate transition zone, initiating microvoids that coalesce into macroscopic debonding paths [9]. Furthermore, curing is neither isothermal nor uniform: dielectric analysis (DEA) and differential scanning calorimetry (DSC) reveal that variable-temperature curing induces spatial heterogeneity in crosslink density, where localized vitrification restricts chain mobility before full conversion, resulting in modulus gradients that concentrate stress at phase boundaries [10]. However, fundamental studies on adhesion thermodynamics, cure–kinetic–structure–property relationships, and thermo-mechanical interfacial stability remain fragmented and insufficiently documented.

To address these issues, this study aims to develop a toughened epoxy adhesive system that exhibits rapid curing, high toughness, low shrinkage, and strong adhesion under ambient conditions. Subsequently, bauxite clinker, a resource-abundant aggregate with excellent skid resistance, was selected as the anti-skid aggregate to construct a highly skid-resistant toughened surface treatment system and implement it in practical application.

## 2. Materials and Methods

### 2.1. Raw Materials

The epoxy resins used in this study include Phoenix brand bisphenol A epoxy resin E51 (Wuxi, China), epoxy resin TDE-85 (Wuhan, China), and Nanya brand bisphenol F linear epoxy resin NPEF170 (Shenzhen, China). Their technical specifications are shown in Table 1.

Cardanol was produced by Jining Huakai Resin Co., Ltd., Jining, China. It is a yellowish-brown liquid with a purity of 98%, density (30 °C) of 0.9296 g/cm^3^, and viscosity (30 °C) of 38 mPa·s.

TETA was produced by Tianjin Komiou Chemical Reagent Co., Ltd., Tianjin, China. It is a pale-yellow viscous liquid with purity ≥98% and density (20 °C) of 0.9770 g/cm^3^.

BDDE was produced by Jining Baiyi Chemical Co., Ltd., Jining, China. It is a colorless transparent liquid with purity ≥98%, epoxy value of 0.74~0.83 eq/100 g, epoxy equivalent of 120~137 g/eq, and viscosity (25 °C) of 10~20 mPa·s.

The formaldehyde solution is industrial-grade formaldehyde, a colorless transparent liquid with a density (−20 °C) of 0.815 g/cm^3^.

Epoxy-terminated polyurethane (EPU) prepolymer from Jining Zhongjian Chemical Co., Ltd. in Jining, China, was used as a modifier. It is a transparent viscous liquid with a solid content of ≥99%, an epoxy equivalent weight of 1100 g/eq, and a viscosity of 30,000–50,000 mPa·s. The specified epoxy equivalent weight indicates that the PU chains are terminated with epoxy groups, enabling their chemical incorporation into the curing network. Based on its application in outdoor, anti-yellowing coatings, it is inferred to be an aliphatic PU prepolymer.

### 2.2. Sample Preparation

#### 2.2.1. Synthesis of TETA-Modified Cardanol Curing Agent (Curing Agent A)

Using cashew phenol, triethylenetetramine (TETA), and formaldehyde (F) as raw materials, the cashew phenol modified triethylenetetramine curing agent was synthesized via the Mannich reaction. The test procedure is as follows.

First, cashew phenol reacts with formaldehyde to form a monomer. Then, the active hydrogen on the amine group of triethylenetetramine undergoes condensation polymerization with the hydroxymethyl group of the cashew phenol formaldehyde, introducing the basic triethylenetetramine monomer unit into the main chain structure of the cashew phenol formaldehyde, thereby obtaining the cashew phenol modified triethylenetetramine curing agent [11].

#### 2.2.2. Synthesis of BDDE Toughening-Modified Mannich Base Curing Agent (Curing Agent B)

**Figure 2:**
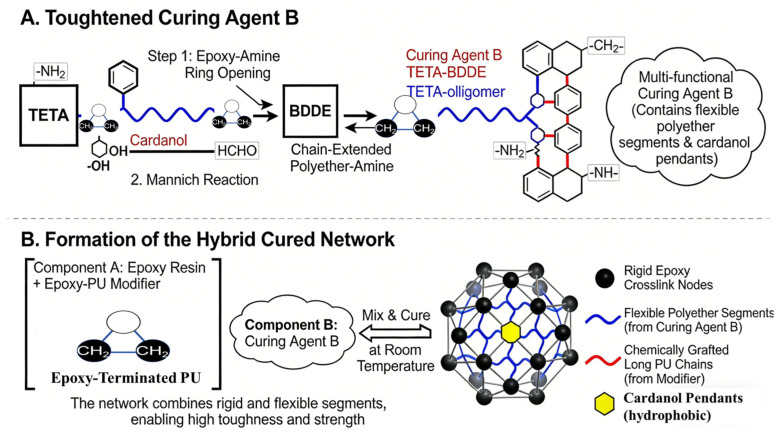
Schematic illustration of the synthesis and cured network formation.

Using a long-chain aliphatic amine (TETA) as the matrix, a flexible-segment-containing room temperature curing agent was synthesized via a two-step strategy: first, chain extension via epoxy-amine addition to build a flexible backbone; second, functionalization via a Mannich reaction to introduce hydrophobic cardanol groups and finalize the multi-amine structure. The conceptual reaction pathway is summarized in Figure 2, and the detailed procedure was as follows.

(1) Addition Reaction (Chain Extension of TETA with BDDE)

A measured amount of triethylenetetramine (TETA) was charged into a four-necked flask and heated to 65 °C under stirring. At this temperature, 1,4-butanediol diglycidyl ether (BDDE) was added dropwise. This step involves the ring-opening addition of the epoxy groups of BDDE to the primary amine groups of TETA, thereby grafting flexible polyether segments into the amine backbone. The reaction was maintained at 65 °C for 3 h to obtain the chain-extended amine intermediate, which was then set aside for the next step.

(2) Mannich Reaction (Synthesis of the Final Curing Agent)

This step was conducted in a three-necked flask equipped with a stirrer, thermometer, and condenser. First, cardanol was mixed with a measured amount of formaldehyde solution (F). The mixture was heated to 80–90 °C and held at this temperature for 50 min to promote the formation of methylolated cardanol intermediates. Next, the amine intermediate from step (1) was added dropwise, and the temperature was raised to 90 ± 5 °C. The Mannich condensation then proceeded, where the amine groups attacked the electrophilic methylolated cardanol, forming stable-CH_2_- bridges and incorporating cardanol as pendant groups. The reaction was carried out under reflux for a period, followed by further reaction for 60 to 120 min. Finally, water was removed from the system by vacuum distillation, and the product was cooled to room temperature to yield the cardanol-modified, flexible-segment-containing epoxy curing agent (Curing Agent B).

#### 2.2.3. Preparation of EPU Prepolymer-Modified Epoxy Resin

The EPU prepolymer (with terminal epoxy groups, as detailed in Section 2.1) was chemically incorporated into a base epoxy resin to produce the modified Component A. Specifically, the base epoxy resin was first preheated to 70 °C, followed by the dropwise addition of the EPU prepolymer at a designated ratio. The reaction was allowed to proceed for 2.5 h under continuous stirring. Thereafter, a catalyst was introduced, and the reaction continued for an additional 1 h, yielding the final polyurethane-modified epoxy resin.

#### 2.2.4. Preparation of BDDE Toughening-Modified Mannich Base Epoxy System

As a two-component system, the EPU prepolymer-modified epoxy resin serves as Component A, and the Curing Agent B from Section 2.2.3 serves as Component B. By mixing Components A and B uniformly under room temperature conditions, the BDDE toughening-modified Mannich base epoxy system can be obtained.

### 2.3. Test Methods

In this manuscript, any numerical results obtained through measurement that may vary due to material inhomogeneities or operational fluctuations (such as toughness, strength, content, viscosity, etc.) were performed in triplicate/quintuplicate parallel experiments, and the results are expressed as the mean or mean ± standard deviation.

(1) Amine Value

Determined using the HCl-ethanol method, and the amine value was calculated according to Equation (1).

X = (C × V × 56.1)/m(1)

where X is the amine value (mg/g); C and V are the concentration (mol/L) and consumption volume (mL) of the HCl-ethanol standard solution, respectively; and m is the sample mass (g).

(2) Viscosity

Measured according to the GB/T 2794-2024 [12] standard using a Changji NDJ-8S rotational viscometer (Shanghai, China) at a test temperature of 25 °C.

(3) Mechanical Property Testing

Tensile, compressive, flexural and impact property tests were conducted in accordance with GB/T 2567-2021 [13]. All tests were performed at 25 °C on a UTM5000 universal testing machine manufactured by Sansi Zongheng (Shenzhen, China).

(4) Bonding Strength

The bonding strength between the epoxy resin and asphalt substrate was measured using the HC-V10S pull-out tester from Haichuang Gaoke Technology Co., Ltd., Beijing, China. Marshall specimens were used to simulate the asphalt substrate for the test. The substrate surface was first cleaned, after which the adhesive was applied; the bonding strength test was then conducted once the adhesive had fully cured.

(5) Curing Process

To characterize the curing rate of the epoxy resin system at different temperatures, 0 °C, 25 °C and 60 °C were chosen as the test temperatures, with the tests carried out in accordance with the procedures below.

① Pot Life: Prepare 4 g of the epoxy adhesive. Timing commences at the instant the epoxy components are uniformly mixed and ends when the adhesive viscosity rises significantly, rendering it difficult to apply evenly onto the bonded substrates.
② Gel Time (Technical requirement: ≥10 min): Prepare 4 g of the epoxy adhesive. Timing starts at the instant the epoxy components are uniformly mixed and terminates when the adhesive viscosity surges sharply, the adhesive can no longer be easily stirred with a glass rod, and a “stringing” phenomenon is observed.
③ Hardening Time: Prepare 4 g of the epoxy adhesive. Timing initiates at the instant the epoxy components are uniformly mixed and finishes when the adhesive surface is no longer tacky, and the adhesive sample undergoes brittle failure or fracture upon the application of a moderate stress.

(6) Fourier Transform Infrared Spectroscopy (FTIR)

Spectroscopy was employed to characterize the functional groups of the samples before and after modification in accordance with GB/T 6040-2019 [14], with a spectral scanning range of 4000–500 cm^−1^. The measurements were performed on a Nicolet 6700 FTIR spectrometer (Thermo Fisher Scientific, Waltham, MA, USA), and all test samples were prepared using the KBr pellet pressing method.

(7) Scanning Electron Microscopy (SEM)

Scanning electron microscopy (SEM) was employed to observe the tensile fracture surfaces of cured epoxy resin specimens for the analysis of fracture modes. All specimens were sputter-coated with a thin gold layer prior to microscopic observation. The tests were performed on a Hitachi TM4000 III benchtop scanning electron microscope (Hitachi, Tokyo, Japan).

(8) Thermogravimetric Analysis (TG)

Thermal stability tests were carried out in accordance with GB/T 33047.1-2016 [15]. A dried sample of 5–10 mg was placed in a crucible and heated from 30 °C to 800 °C at a heating rate of 10 °C/min under a nitrogen atmosphere, with the thermogravimetric (TG) curve recorded throughout the process. Thermogravimetric measurements were performed on a Netzsch TG209 thermogravimetric analyzer (Netzsch, Selb, Germany).

(9) Paper Cup Test

Two groups of 100 g each of flexible curing agent-epoxy resin adhesive systems with the optimal process and dosage were prepared. At the same time, three groups of 500 g each of epoxy-curing adhesive systems with added bauxite clinker were prepared. They were placed horizontally at room temperature (25 °C) and high temperature (60 °C), and empty paper cups were used as references. After the systems were fully cured, the deformation degree of the paper cups was observed to intuitively characterize the thermal sensitivity of the epoxy adhesive systems.

(10) Macroscopic Cracking Characterization

First, prepare the epoxy resin adhesive system; the cracking situation of the epoxy adhesive system and the underlying layer (asphalt pavement) was preliminarily characterized through repeated low-temperature (60 °C oven) and high-temperature (freezer) curing, and the specific process is as follows:

① Prepare a rutting board according to the specification requirements to simulate an asphalt pavement;
② Divide the rutting board evenly into two parts with tape, one half evenly coated with an appropriate amount of epoxy resin adhesive and one part as a blank reference;
③ After the above epoxy adhesive is fully cured, place the rutting board in an oven set to 60 °C for 12 h, then place it in the refrigerator for 12 h and repeat the cycle. Regularly observe and record the surface condition of the rutting board and take photos.

(11) Nuclear Magnetic Resonance (NMR)

^1^H-NMR and ^13^C-NMR spectra were obtained using deuterated chloroform as the solvent. The instrument used was a Bruker (Rheinstetten, Germany) Avance III 400 MHz NMR spectrometer. Mnova V15.0 NMR analysis software was used for mapping and analysis.

(12) Linear Expansion Coefficient Test

The tests were conducted over a temperature range of −30 °C to 100 °C at a heating rate of 5 °C/min. The test specimens were prepared to dimensions of 30 mm ×10 mm × 2 mm (length × width × thickness). All measurements were performed using a Zhonglu ZRPY-DW low-temperature thermal expansion coefficient tester (Beijing, China).

## 3. Results and Discussion

### 3.1. Synthesis Process and Optimization of Proportions of Curing Agent A

#### 3.1.1. Study on the Effect of Raw Materials on the Performance of Curing Agent A

The amine value and viscosity of the synthesized curing agent were the main evaluation indicators. The optimal time and temperature for synthesis were determined using a single-factor experimental method, and the optimal cashew phenol content and triethylenetetramine content were selected.

(1) Triethylenetetramine dosage

**Figure 3:**
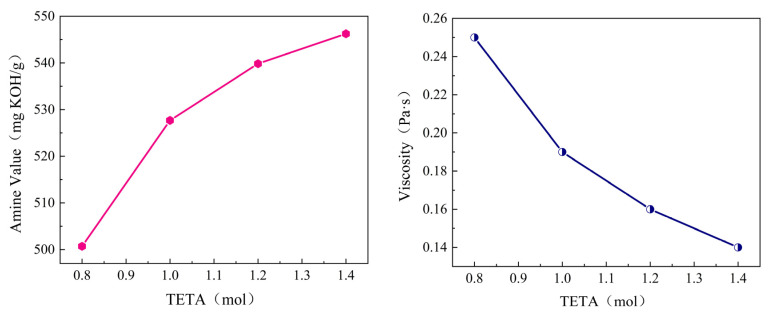
Effect of TETA dosage on the amine value and viscosity of the system.

To determine the optimal dosage of TETA, curing agents with different TETA dosages were synthesized (other raw material ratios were referenced from the manufacturer’s recommended values). By testing the amine value and viscosity indicators, the optimal dosage was determined. The experimental results are shown in Figure 3.

As shown in Figure 3, with the increase in TETA dosage, the amine value of the curing agent rose gradually while its viscosity decreased steadily. At a fixed cardanol molar amount of 1 mol, elevating the TETA dosage from 0.8 mol to 1.4 mol caused the rate of increase in amine value to slow down progressively, an indication that the reaction was approaching completion. At the same time, the proportions of cardanol and formaldehyde (F) in the reaction system declined, which reduced the number of reactive sites available for the formation of macromolecular products and thus led to a lower viscosity of the curing agent.

Taking both the amine value and viscosity of the product into account, a TETA dosage of 1.4 mol was selected for all subsequent experiments. This is because at a molar ratio of cardanol to TETA of 1:1.4, the curing agent achieved a near-maximum amine value, which allows for the minimum dosage of curing agent when blended with epoxy resin (EP). A low amine value would not only compromise the curing efficiency of EP but also result in higher application costs. In addition, excessively high viscosity of the curing agent would lead to poor self-leveling and wetting performance, hindering uniform mixing with EP and potentially causing curing defects such as partial or incomplete curing.

(2) Cashew phenol dosage

**Figure 4:**
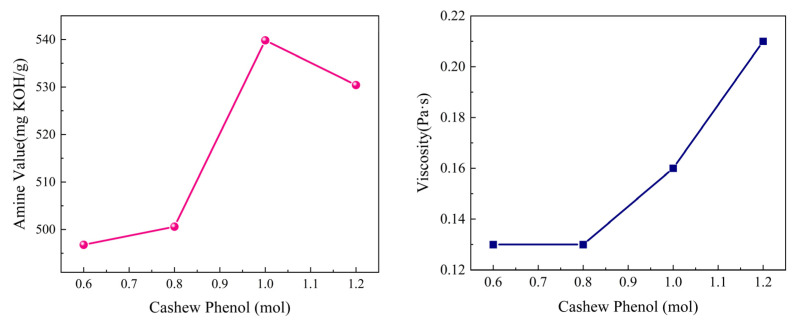
Effect of cashew phenol dosage on the amine value and viscosity of the system.

To determine the optimal dosage of cashew phenol, curing agents with different cashew phenol dosages were synthesized (the TETA dosage was fixed at 1.4 mol, and the ratio of other raw materials remained unchanged). By testing the amine value and viscosity indicators, the optimal dosage was determined. The experimental results are shown in Figure 4.

As shown in Figure 4, with the increase in cashew phenol dosage, the amine value of the curing agent system first increases and then decreases, while the viscosity gradually increases. Based on a comprehensive consideration of amine value and viscosity, the dosage of cashew phenol was selected as 1.0 mol in the subsequent study.

The reason for the above phenomenon is that the ortho groups on the benzene ring in cashew phenol participate in the condensation reaction, consuming part of the triethylenetetramine, which leads to a decrease in the amine value. In addition, due to the presence of the C15 long-chain substituent in cashew phenol, the distance between the double bonds is short, showing a significant electronic effect (relatively low reactivity), and the large steric hindrance forms a relatively tight spatial structure, increasing the viscosity of the product [16].

#### 3.1.2. Study on the Effect of Synthesis Process on the Performance of Curing Agent A

(1) Reaction time

**Figure 5:**
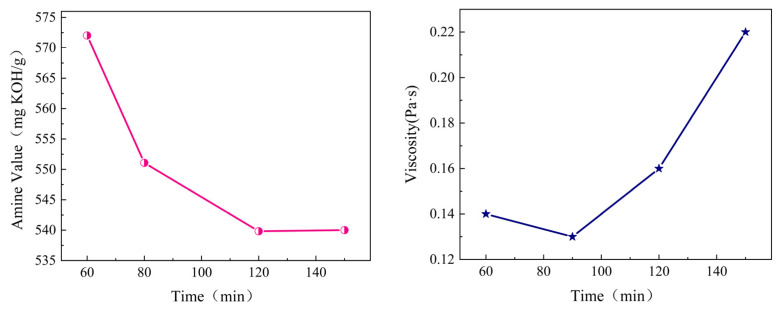
Effects of reaction time on the amine value and viscosity of the curing agent system.

To determine the optimal reaction time, the effects of different reaction times on the amine value and viscosity of the curing agent system were studied. The experimental results are shown in Figure 5.

As presented in Figure 5, the amine value of the reaction system decreased gradually with prolonged reaction time, whereas the viscosity exhibited a trend of first increasing and then decreasing. Based on a comprehensive consideration of amine value and viscosity, the optimal reaction time for the system was set at 120 min for subsequent investigations.

Extending the reaction time from 60 min to 120 min led to a marked decrease in the system’s amine value. Thereafter, further prolongation of the reaction time resulted in a gradual reduction in the rate of amine value change, demonstrating that the reaction within the system had essentially reached completion by the 120 min mark. The variation in the curing agent’s viscosity is attributed to the condensation reaction of specific functional groups during the later stages of the reaction.

(2) Reaction temperature

**Figure 6:**
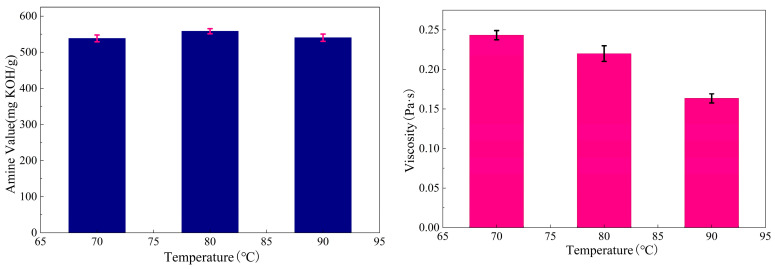
Effects of temperature on the amine value and viscosity of the curing agent system.

To determine the optimal reaction temperature of the system, the effects of different synthesis temperatures on the amine value and viscosity of the curing agent system were studied. The experimental results are shown in Figure 6.

As illustrated in Figure 6, elevating the reaction temperature from 70 °C to 90 °C caused the system’s amine value to remain nearly unchanged while the viscosity decreased steadily. By balancing amine value and viscosity, the reaction temperature was fixed at 80 °C for all subsequent experiments.

This phenomenon can be explained by the fact that elevated temperatures enhance the flexibility of the long cardanol molecular chains, which in turn reduces the number of physical cross-linking points formed by intermolecular hydrogen bonds. This facilitates molecular movement at room temperature and thus leads to a reduction in viscosity. In addition, the number of amino groups remains constant upon reaction completion, and no thermal decomposition takes place under the tested temperature range, resulting in only a slight variation in amine value.

### 3.2. Material Composition Design and Performance Characterization of Curing Agent B

#### 3.2.1. Synthesis Process and Optimization of Proportions of Curing Agent B

Based on the above findings, with the optimal molar ratio of cardanol:TETA:formaldehyde (F) set at 1:1.4:0.8, the optimal raw material proportions and reaction conditions for the system were further determined by taking viscosity and amine value as key evaluation indicators, with the corresponding test results presented in Table 2, Table 3 and Table 4.

An analysis of Table 2, Table 3 and Table 4 reveals that the optimal reaction temperature and time for the system are 65 °C and 3 h, respectively. Under these optimal conditions, the curing system exhibits a moderate viscosity and a favorable amine value. In addition, the system’s viscosity shows a trend of first increasing and then decreasing with the rise in BDDE dosage, while the amine value decreases continuously throughout this process. A comprehensive analysis led to the determination of an optimal TETA-to-BDDE molar ratio of 2:1 for the present study.

This phenomenon can be attributed to two main factors. First, during the chain extension stage, an increase in BDDE dosage leads to the formation of longer molecular chains with higher molecular weights and stronger intermolecular forces, consequently increasing the system’s viscosity. However, excessive BDDE dosage results in the near-saturation of amino groups consumed in the reaction, leading to the presence of unreacted low-viscosity BDDE in the system. This unreacted BDDE does not participate in the chain extension reaction but instead acts as a diluent to reduce the overall viscosity of the system. Second, the epoxy groups of BDDE continuously consume the amino groups on TETA molecules with increasing BDDE dosage, and the proportion of low-molecular-weight, nitrogen-free BDDE components in the system rises accordingly. This causes a physical dilution of the effective concentration of amino groups per unit sample, leading to a gradual decrease in amine value.

By integrating the experimental results from Section 3.3.1 and Section 3.3.2, a mass ratio of epoxy resin (EP) to Curing Agent B of 1:1 was selected for all subsequent experiments.

#### 3.2.2. Performance Characterizations of Curing Agent B

(1) Infrared spectrum (FTIR)

**Figure 7:**
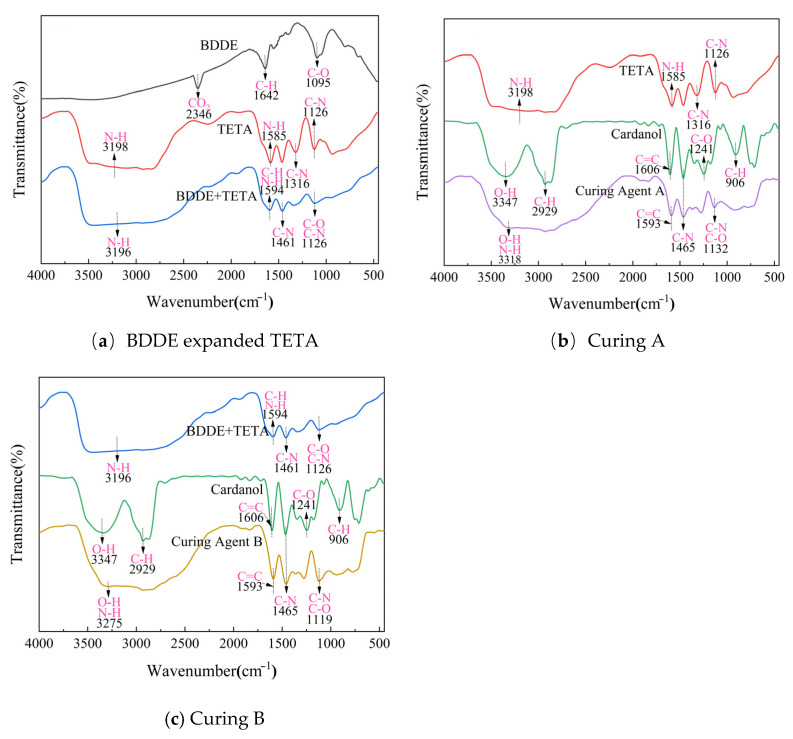
Infrared spectrum.

The FTIR spectroscopy results are shown in Figure 7, and the spectral changes and their assignments are presented in Table 5, Table 6 and Table 7.

Figure 7a and Table 5 present the infrared spectra and corresponding spectral analysis data for pure TETA, a TETA-BDDE physical mixture, and BDDE-chain-extended TETA. The results prove that the aliphatic chain and ether bond structure inside the BDDE have been successfully grafted into TETA.

First, the infrared characteristic peaks of BDDE are relatively simple, showing only C-H and C-O bond infrared characteristic peaks at 1642 cm^−1^ and 1095 cm^−1^, which come from the aliphatic carbon chains and ether bonds inside the sample. After chain extension, the FTIR curve of the sample is still the same as that of TETA, but the wavelength of the key characteristic peaks has shifted. The original N-H characteristic peak at 1585 cm^−1^ has shifted to 1594 cm^−1^ under the influence of the aliphatic chain in the diluent, showing an overlapped infrared peak of C-H and N-H bonds. At the same time, the C-O bond at 1126 cm^−1^ is retained [17].

This result proves that Curing Agent B has been successfully synthesized and the reaction is chemical. Figure 7b and Table 6 display the infrared spectra and related analysis results for cardanol, pure TETA, and Curing Agent A. These results confirm the successful synthesis of Curing Agent A and verify that a chemical reaction rather than a physical mixing process occurred during its preparation.

First, the changes in the O-H and N-H bonds at 3318 cm^−1^ are relatively obvious. The broad peak of N-H in TETA is retained, but the peak shape changes under the influence of the phenolic hydroxyl group in cardanol, which is key evidence of their synthesis. In addition, Curing Agent A also shows characteristic peaks of C=C and C-N bonds at 1593 cm^−1^ and 1465 cm^−1^, respectively. The unchanged wavelength in the amide II band (C-N) indicates the stable existence of cardanol. In addition, Curing Agent A also shows infrared characteristic peaks of C-N and C-O bonds at 1132 cm^−1^, while the characteristic peaks of original TETA and cardanol at 1126 cm^−1^ and 1241 cm^−1^ disappear [18].

Figure 7c and Table 7 report the infrared spectra and corresponding analysis data for cardanol, BDDE-chain-extended TETA, and Curing Agent B. The reaction process is similar to that of Curing Agent A, and the FTIR curve trends of the two are roughly similar, but the wavelengths of the O-H bond, N-H bond (3275 cm^−1^), and C-O bond (1132 cm^−1^) differ. This is because the chain-extended TETA has increased phenolic hydroxyl and ether bonds, which result in different group vibrations when reacting with cashew phenol, although the basic structure is the same.

(2) Nuclear magnetic resonance (NMR)

To further understand the structures of the self-made Curing Agent A and B, nuclear magnetic resonance analysis (carbon spectrum, hydrogen spectrum) was performed. The test results are as follows.

**Figure 8:**
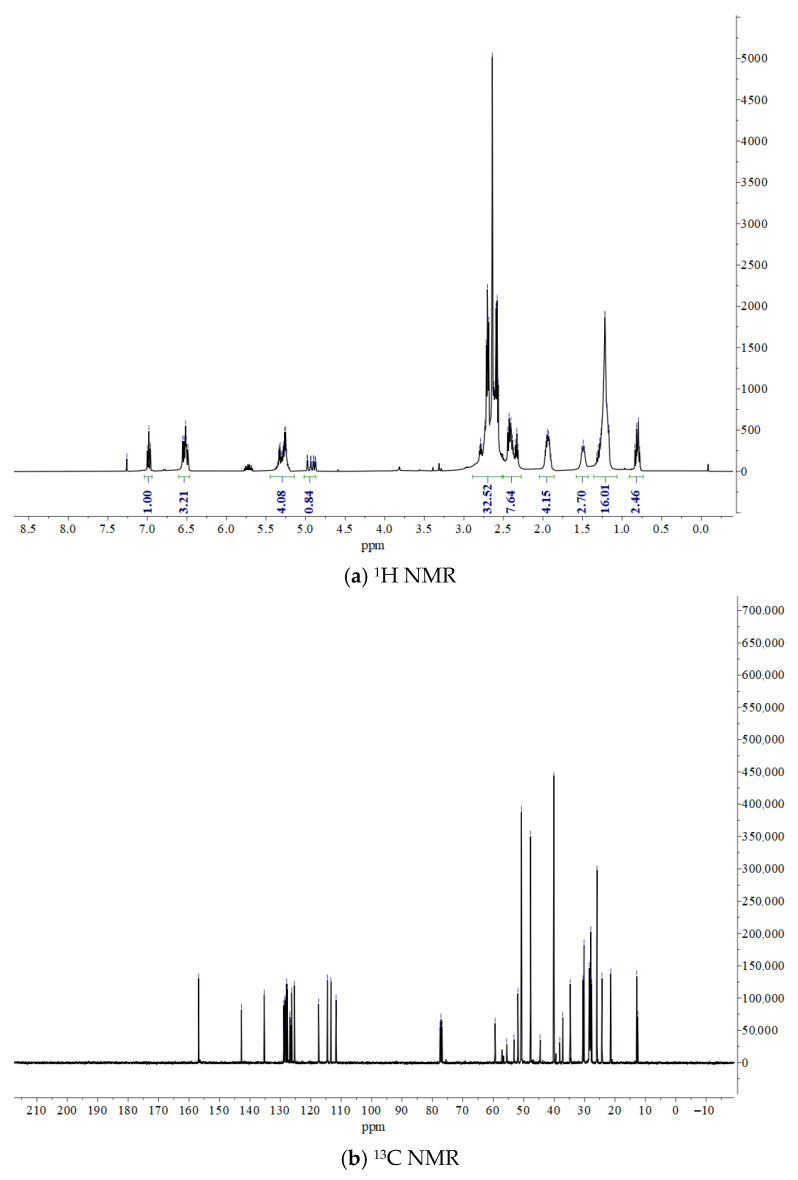
NMR test results for Curing Agent A.

Combining the results from Figure 8 and Table 8 and Table 9, it can be seen that Curing Agent A is a mixture of Mannich base oligomers produced by the reaction of triethylenetetramine and cardanol via the Mannich reaction. Firstly, the key signals in the carbon spectrum in the 40–55 ppm range are direct evidence of the Mannich base structure. Secondly, both the hydrogen and carbon spectra retain the characteristic signals of cardanol and TETA. Furthermore, peak broadening and multiple signals indicate that the product is not a single compound, consistent with the actual situation for this type of hardener [19].

In summary, this compound is a triethylenetetramine-cardanol Mannich base, a mixture of multiple structurally analogous compounds. The amine groups in TEAT undergo the Mannich reaction with formaldehyde and the benzene ring of cardanol, forming an Ar-CH_2_-N< bridge structure. Since TETA has 4 active N atoms, it can link 1–4 cardanol units, resulting in the characteristic superposition of multiple signal groups in the spectra.

**Figure 9:**
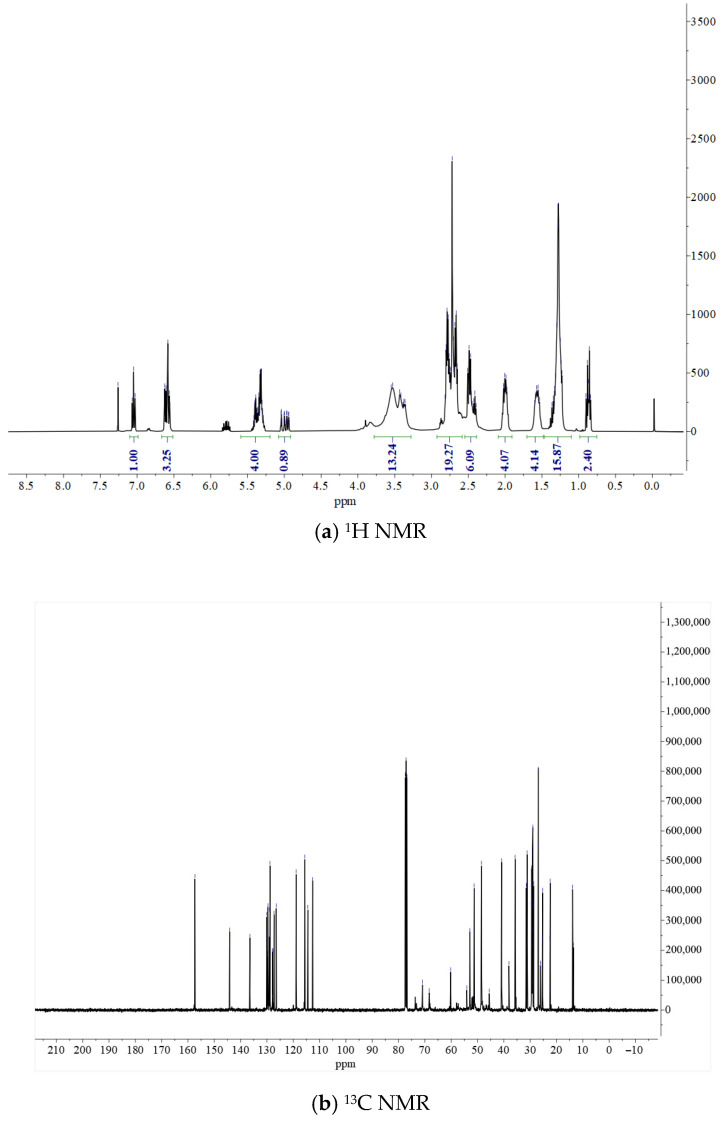
NMR test results for Curing Agent B.

From the test results in Figure 9 and Table 10 and Table 11, it can be seen that hardener B is an epoxy hardener prepared by first subjecting triethylenetetramine (TETA) to epoxy-amine ring-opening polymerization chain extension with 1,4-butanediol diglycidyl ether (BDDE), followed by a Mannich reaction with cardanol. The product is a complex mixture (oligomer/polymer) containing cardanol groups, polyether amine segments, and Mannich base linking groups.

Cardanol Unit: In the hydrogen spectrum: δ 7.05 ppm (aromatic H), δ 5.35–4.86 ppm (olefinic H) and δ 0.87 ppm (terminal CH_3_). In the carbon spectrum: δ 157.6 ppm (phenolic carbon), δ 144.4–112.9 ppm (aromatic and olefinic carbons) and δ 31.7–22.6 ppm (long-chain alkyl carbons). This indicates that the cardanol structure is fully retained, with multiple double bonds in the side chain (the spectrum shows about 5 olefinic protons, possibly a mixture of diene/triene).

Polyether Amine Chain Extension Segment: A broad envelope at δ 3.76–3.17 ppm in the hydrogen spectrum corresponds to -O-CH_2_- of the polyether chain and -CH-OH generated by epoxy ring opening. In the carbon spectrum, δ 71.1, 68.6 ppm are ether carbons; δ 60.4 ppm may include -CH-OH carbons. This confirms that TETA and BDDE have undergone ring-opening polymerization, forming a polyether amine backbone containing hydroxyl groups.

Mannich Base Linker: In the hydrogen spectrum, part of the signal at δ 3.76–3.17 ppm and the broad envelope at δ 2.93–2.60 ppm contain Ar-CH_2_-N hydrogens (typically found at δ 3.8–4.2, shifted upfield here due to environmental overlap). The multiple signals in the carbon spectrum in the δ 54.3–45.8 ppm range are typical of Mannich base carbons (-Ar-CH_2_-N<) and carbons adjacent to amines. This indicates that cardanol is attached via a Mannich reaction through a -CH_2_- bridge to the nitrogen atoms of the polyether amine [20].

In summary, the characteristics of this mixture are as follows: The total integral in the hydrogen spectrum is much larger than that of a single small molecule, with significant signal broadening and overlap (especially in the δ 2.5–4.0 region). The carbon spectrum shows numerous closely spaced signals (e.g., multiple methylene carbons around 29.7–29.2 ppm), reflecting differences in cardanol side chain unsaturation, polyether amine chain length distribution, and linking mode diversity. That is, the ^1^H NMR and ^13^C NMR data fully conform to the expected structure of hardener B, clearly displaying the characteristic signals of cardanol, the polyether amine chain extension segment, and the Mannich base linker. The spectral complexity arises from the oligomeric/polymeric mixture nature of the product, which is typical for this class of epoxy hardeners.

### 3.3. Performance Characterization of BDDE Toughening-Modified Mannich Base Epoxy System

#### 3.3.1. The Effect of Curing Agent B on the Toughness of Epoxy Resin Curing Systems

**Figure 10:**
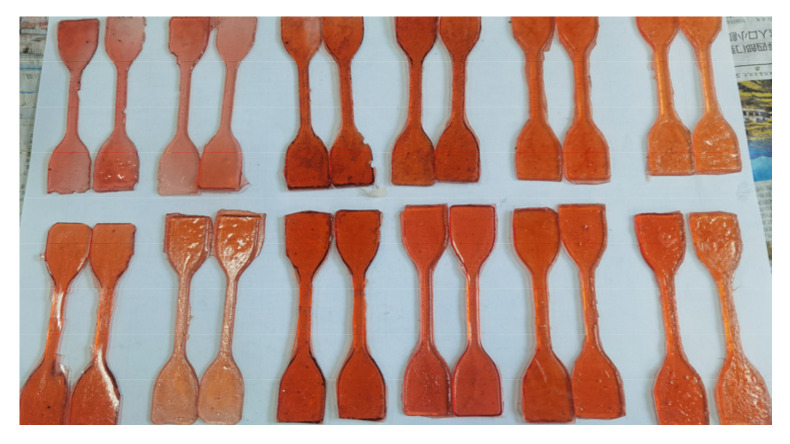
Tensile sample diagram.

Figure 10 and Table 12 show the tensile test specimens and the corresponding test results for epoxy resin cured products with different mass ratios, following 7 days of storage at room temperature (25 °C).

As shown in Table 12, with the increase in the amount of Curing Agent B, the elongation at break of the epoxy resin cured product increases, while the tensile strength decreases. When the ratio of epoxy resin to curing agent is 1:1, the elongation at break of the epoxy curing system reaches its maximum value of 40.41%, with a tensile strength of 7.12 MPa. This indicates that Curing Agent B can effectively improve the toughness of the epoxy resin curing system.

#### 3.3.2. The Effect of Curing Agent B on the Bonding Strength of Epoxy Resin Curing Systems

**Figure 11:**
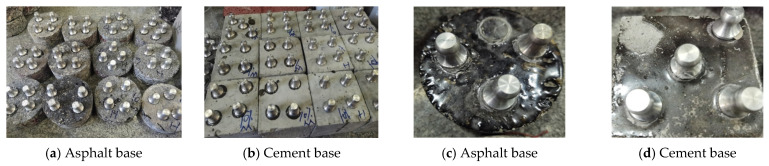
(**a**,**b**) are photographs of the adhesion strength test of the epoxy adhesive system; (**c**,**d**) are conditions of the cement and asphalt bases after pull-off testing.

Table 13 shows the adhesive strength of the epoxy resin system with different ratios under room temperature conditions (25 °C). The adhesive strength test process and results are shown in Figure 11 and Table 13, respectively.

From Table 13, it can be seen that as the amount of Curing Agent B increases, the adhesion strength between the epoxy resin and both types of substrates also increases. At room temperature (25 °C), when the mass ratio of epoxy resin to curing agent is 1/1, the pull-out strengths of the epoxy resin on the asphalt substrate and the cement substrate are 6.83 MPa and 8.07 MPa, respectively. Moreover, the adhesion strength of the epoxy resin to the cement substrate is higher than that to the asphalt substrate, demonstrating that the epoxy system exhibits better adhesion to the cement substrate. This is because the interfacial failure mode between the cement substrate and the epoxy resin is cohesion failure within the cement substrate (Figure 11).

Based on the combined results from Section 3.3.1 and Section 3.3.2, the mass ratio of epoxy resin to Curing Agent B for subsequent studies was determined as *m* (EP):*m* (curing agent B) = 1:1.

#### 3.3.3. The Effect of Curing Agent B on the Heat Resistance of Epoxy Resin Curing Systems

**Figure 12:**
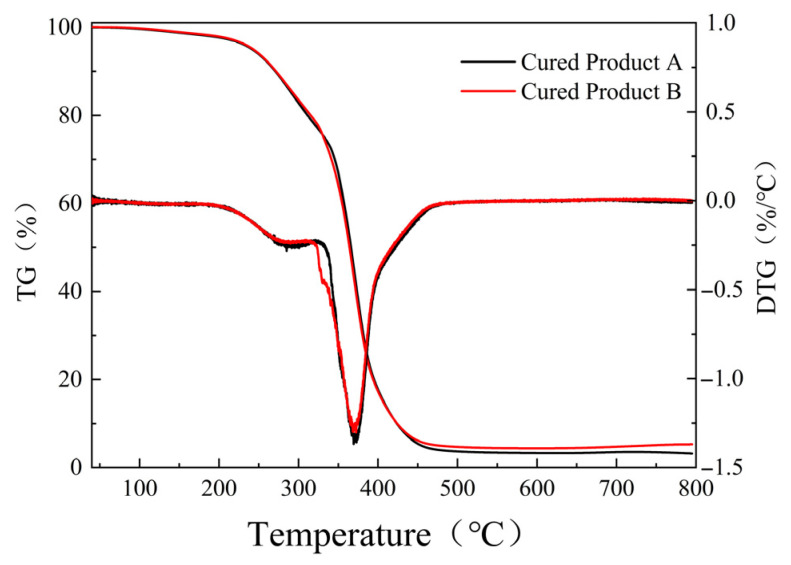
Thermogravimetric analysis results.

In Figure 12, Cured Product A is the cured product of cashew phenol-modified amine epoxy resin curing agent + epoxy resin, and Cured Product B is the cured product of 1,2-butanediol diglycidyl ether and triethylene tetramine chain-extended and then synthesized with cashew phenol epoxy curing agent + epoxy resin.

As can be seen from Figure 12, the first weight loss range of Cured Product A is 30–323 °C (22.6% weight loss, DTG peak temperature 289 °C), which is speculated to occur in two stages: first, weight loss in the 30 °C to 200 °C range is due to volatilization of small molecule residues, such as trace moisture absorbed from the environment and unreacted monomers remaining in the system. Secondly, in the 200 °C to 323 °C range, the main reasons are the cleavage and volatilization of the cardanol long alkyl chain and decomposition of some amine crosslinking points (C-N bonds). This is because the cardanol aliphatic long chain has lower thermal stability, and weak crosslinks in the epoxy-amine system may also break simultaneously. The second weight loss range is 323–800 °C (74.2% weight loss, DTG peak temperature 373 °C), which is speculated to correspond to the decomposition of the main epoxy network, including the cleavage of benzene rings, ether bonds, amine bonds, and deep cracking of the residual aromatic rings [21].

For Cured Product B, the first weight loss range is 30–313 °C (20.3% weight loss, DTG peak temperature 288 °C), and the mechanism is similar to that of Cured Product A: volatilization of small molecule residues, cleavage of the cardanol side chain, and decomposition of some amine bonds. Although the ether bonds introduced by the BDDE increased the network flexibility, they did not change the core decomposition pathway. The second weight loss range is 313–800 °C (74.4% weight loss, DTG peak temperature 370 °C), which is attributed to the decomposition of the main epoxy network. Due to the lower thermal stability of the ether bonds, the decomposition peak temperature is slightly lower than that of Cured Product A [22].

In terms of thermal resistance, the DTG peak temperatures of Cured Product A in both stages (289 °C, 373 °C) are higher than those of Sample 2 (288 °C, 370 °C), especially the second stage which differs by 3 °C, indicating that the main decomposition process of Cured Product A occurs at a higher temperature. In terms of the transition temperatures between stages, the first stage of Cured Product A ends at 323 °C, which is higher than the 313 °C for Cured Product B, showing slightly better thermal stability in the low-temperature range. In summary, the thermal stability of Cured Product A is slightly better than that of Cured Product B. This is mainly attributed to the more compact crosslinking network in Cured Product A, while the increased proportion of ether bonds in Cured Product B reduces the rigidity of the molecular chain, leading to a slight advancement in the thermal decomposition.

#### 3.3.4. The Effect of Curing Agent B on the Micro-Morphology of the Tensile Fracture Surface of Epoxy Resin

**Figure 13:**
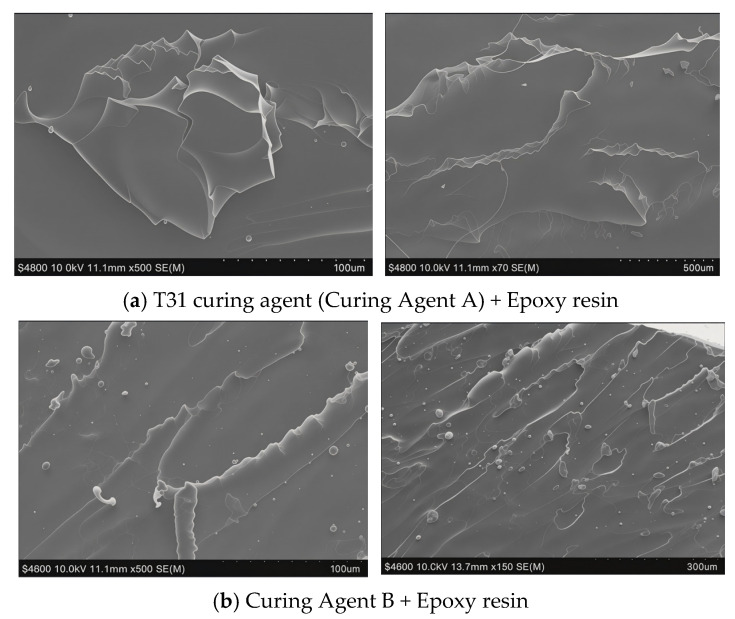
SEM images of the epoxy fracture surface.

The micro-morphology of the fracture surface of different curing agent + epoxy resin systems was observed using a scanning electron microscope. The results are shown in Figure 13:

As depicted in Figure 13, the fracture surface of the T31 (Curing Agent A)-EP cured system is relatively flat and smooth, a typical characteristic of brittle fracture. In contrast, the fracture surface of the self-prepared Curing Agent B-EP cured system is rough and undulating, with localized dimples observed, indicative of ductile fracture. This demonstrates that the BDDE-chain-extended epoxy curing system has superior toughness, which is highly consistent with the macroscopic test results presented in subsequent Section 3.5.1 (1). After chain extension, the elongation at break of the epoxy curing system increased from 28.7% to 40.4%, a rise of 28.9% percentage points.

This improvement in toughness stems from the introduction of flexible segments, which enhances the segmental mobility of the epoxy crosslinked network. When subjected to external stress, the epoxy resin can dissipate energy through chain rearrangement and slippage, thereby preventing the rapid propagation of cracks induced by stress concentration [23].

### 3.4. Study on the Effect of Toughening Agents on the Properties of the Epoxy-Curing System

#### 3.4.1. The Effect of EPU Prepolymer Content on the Mechanical Properties of Epoxy Resin

Based on the above research results, following the method described in Section 2.3 (3), the effect of EPU prepolymer content on the mechanical properties of the epoxy resin curing system was investigated. The test results are shown in Table 14.

From Table 14, it can be seen that as the amount of toughening agent increases, the tensile strength of the epoxy adhesive system first increases and then decreases. When the toughening agent content increases from 5% to 20%, compared to the epoxy adhesive system without the toughening agent, the elongation at break increases from 40.4% to 44.3%, an increase of 8.8%. This indicates that an appropriate amount of toughening agent can effectively improve the tensile flexibility of the epoxy adhesive system. However, when the EPU prepolymer content exceeds 10%, the tensile strength of the epoxy adhesive system shows a slight downward trend. Accordingly, an EPU prepolymer dosage of 10 wt% relative to epoxy resin was selected for all subsequent experiments.

The main reason for this phenomenon is that physical blending and chemical crosslinking reactions occur between the active groups of epoxy resin and EPU prepolymer, forming a crosslinked network structure. At the same time, the microphase separation structure between the two increases with the increase in EPU prepolymer content. Studies show that both the crosslinked network structure and the microphase separation structure can effectively disperse external stress, thereby increasing the tensile strength of the epoxy adhesive system [24]. When the EPU prepolymer content exceeds 10%, the excessive amount of EPU prepolymer leads to over-crosslinking within the epoxy system, or even local aggregation, which adversely affects the tensile properties of the epoxy adhesive system.

#### 3.4.2. The Effect of Toughening Agent Type on the Mechanical Properties of Epoxy Resin

**Figure 14:**
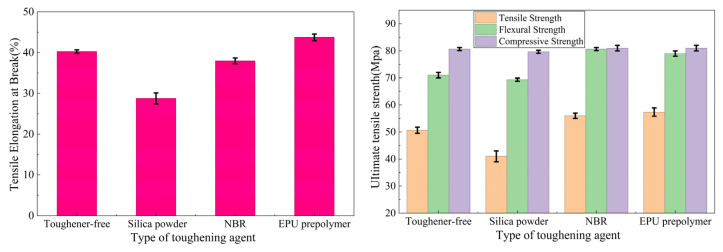
Mechanical properties study of EPU prepolymer-modified epoxy resins.

The elongation at break, tensile strength, flexural strength, and compressive strength of EPU prepolymer-modified epoxy resins were tested in comparison with conventional toughening agents added to the epoxy resin system. The results are shown in Figure 14 (NBR is nitrile butadiene rubber in the picture).

As shown in Figure 14, the EPU prepolymer-modified epoxy resin system has the highest elongation at break, reaching 44.3%, which is an increase of 8.8% compared to the epoxy resin system without a toughening agent. The mechanical properties of the EPU prepolymer-modified epoxy resin system are good, indicating that EPU prepolymer can improve the toughness of epoxy resins without reducing their mechanical properties.

#### 3.4.3. The Effect of the Type of Toughening Agent on the Micro-Morphology of the Tensile Fracture Surface of Epoxy Resins

**Figure 15:**
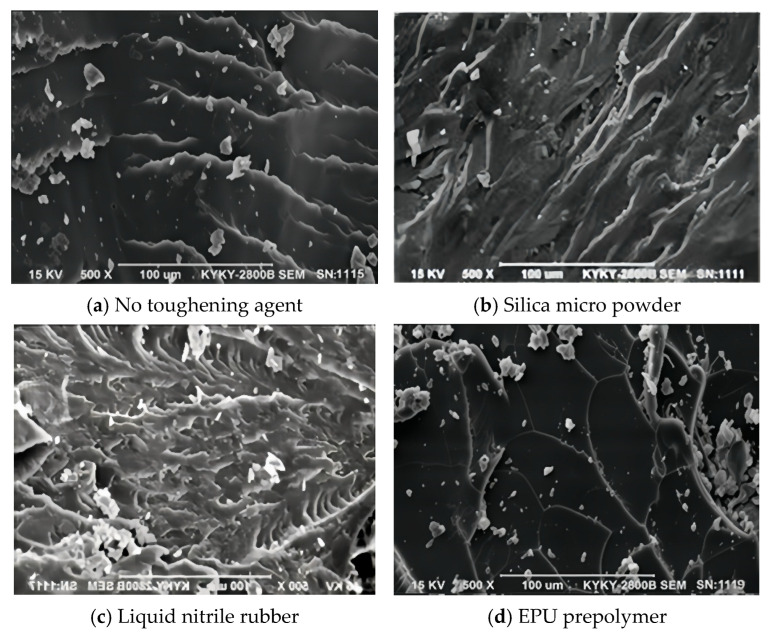
SEM morphology of the tensile fracture surface of epoxy resins.

The micro-morphology of the fracture surface of the epoxy resin system with different toughening agents was observed using a scanning electron microscope. The results are shown in Figure 15:

As shown in Figure 15, the tensile fracture surface of the EPU prepolymer-modified epoxy resin is a typical ductile fracture surface, confirming that the incorporation of EPU prepolymer can effectively improve the toughness of EP. This finding is consistent with the results in Figure 14, which show that the EPU prepolymer-modified epoxy resin achieved the optimal toughness performance.

The SEM micrographs reveal that the fracture surface of the epoxy resin without a toughening agent is smooth, with sharp edges and a consistent fracture direction that tends to continue to propagate, which is a typical brittle fracture; after adding silica micro powder and terminal carboxyl liquid nitrile butadiene rubber, the epoxy resin matrix undergoes plastic deformation, causing energy dissipation and enhancing the ability of the epoxy adhesive to resist external forces. The terminal carboxyl liquid nitrile butadiene rubber has good compatibility with the epoxy resin, enhancing the synergistic effect between the epoxy resin and the toughening agent molecules, making the direction of the fracture crack tend to disperse, and is accompanied by obvious micro-cracks and fish-scale-like structures; after adding the EPU prepolymer, the tensile fracture surface of the epoxy resin matrix shows a distinct shell-like pattern and produces many root-like branches, and the fracture stripes tend to disperse, which is caused by shear yielding of the specimen, showing a relatively obvious ductile fracture feature.

### 3.5. Application Technology Research

#### 3.5.1. Indoor Comparative Test Study

**Figure 16:**
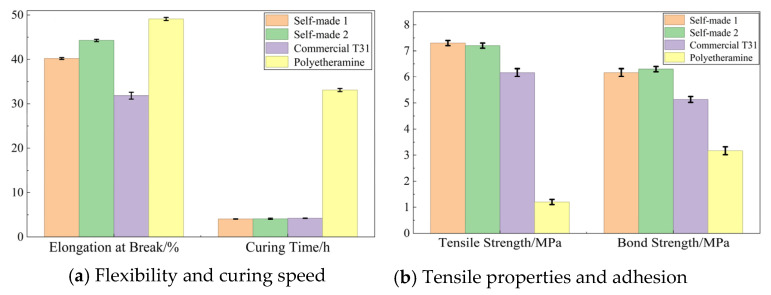
Performance comparison results of different epoxy adhesive systems.

In this section, the performance of the self-prepared curing agents is compared with that of commercially available curing agents. The formulations tested are defined as follows: Self-made 1 (Curing Agent A blended with EP), Self-made 2 (Curing Agent B blended with EP), and Self-made 3 (Self-made 2 with the addition of an EPU prepolymer toughener). All self-prepared formulations were tested at their optimal ratios, while commercial curing agents were used in accordance with the manufacturers’ recommended dosages. The relevant test results are presented in Figure 16 and Table 15, Table 16, Table 17 and Table 18.

(1) Mechanical properties

As can be seen from Table 15, compared with Self-made 1, the fracture elongation rate of the Self-made 2 epoxy adhesive system is 40.4%, an increase of 28.9%, indicating that the toughening modification of the glycidyl ether toughening agent prepared by the chain extension method has a good toughening effect on the epoxy adhesive system.

Compared with Self-made 2, the fracture elongation rate of Self-made 3 is 44.3%, an increase of 8.8%, and the tensile strength reaches 7.0 MPa, indicating that the addition of the EPU prepolymer toughener has little effect on the mechanical properties of the epoxy adhesive system. In addition, the fracture elongation rate of the commercial polyether amine D400 and D2000 series curing agents is high, but their tensile strength is only 0.7 MPa and 0.4 MPa, making it difficult to use them as adhesive systems in anti-slip surface technology.

Curing speed test results of epoxy systems at different temperaturesConsidering the application scenario, the epoxy resin anti-skid system needs to have the characteristic of rapid curing under low-temperature or room temperature conditions to allow for the timely reopening of traffic after completion. Therefore, this study investigated the curing process of its adhesive system.

Table 16, Table 17 and Table 18 compare the curing performance of different epoxy adhesive systems under low-temperature (0 °C), normal-temperature (25 °C), and high-temperature (60 °C) conditions. From the data in the table, it can be seen that the self-developed epoxy adhesive system has the characteristics of rapid curing and high toughness at normal temperature. The 35 min pot life provides a relatively sufficient construction time for engineering, and the 175 min curing time allows the anti-slip surface to be opened to traffic as soon as possible after paving.

From the data in Figure 16 and Table 15, Table 16, Table 17 and Table 18, it can be seen that the elongation at break of the commercial polyether amine D400 and D2000 adhesive systems is better than that of the self-developed adhesive systems. However, their gel time and curing time are too long to meet the rapid curing requirements in highway engineering, and their tensile strength is too low.

#### 3.5.2. Shrinkage and Cracking Performance

(1) Shrinkage Performance

**Figure 17:**
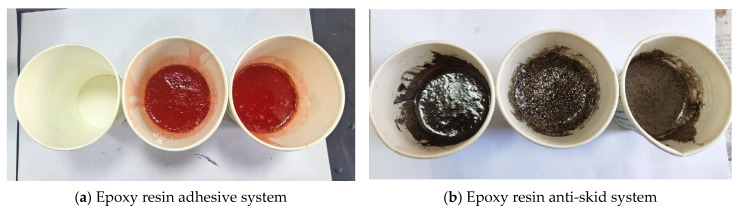
Shrinkage performance test results.

To characterize the shrinkage performance of the epoxy system, tests were conducted according to Section 2.3 (9), and the specific results are shown in Figure 17. In Figure 17a, from left to right, they are the control group, deformation results of the adhesive systems under high-temperature conditions, and room temperature, respectively. In Figure 17b, from left to right, they are the control group, the room temperature condition, and the high-temperature condition of the epoxy–bauxite curing adhesive system deformation results.

From Figure 17, it can be seen that temperature has a certain influence on the shrinkage deformation of the epoxy adhesive system. The deformation degree of the epoxy adhesive system under normal temperature conditions is less than that of the epoxy adhesive system cured under 60 °C conditions.

(2) Thermal expansion property

Research indicates that the molecular structure of epoxy resin is an important factor affecting the temperature shrinkage performance of epoxy adhesive systems [25]. Therefore, this study selected three epoxy resins with different structures: trifunctional cycloaliphatic epoxy resin TDE-85, bisphenol A epoxy resin E51, and bisphenol F linear epoxy resin NPEF170. Combined with the application scenario, their thermal expansion coefficients were tested in the range of −30 °C to 100 °C to determine the type of epoxy resin for subsequent research. In this test, the self-made hardener B was used, and the mixing ratio of epoxy resin to hardener was 1:1 (mass ratio).

**Figure 18:**
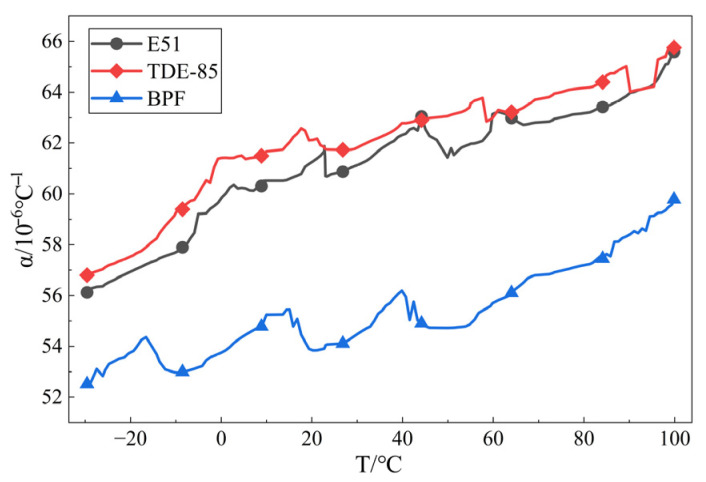
The results of the linear expansion coefficient test.

From Figure 18, it can be seen that as temperature increases, the linear expansion coefficient α of all three epoxy resins generally shows an increasing trend. The α value range for epoxy resin E51 is approximately 56~66 ppm/°C. It is lower in the low-temperature range, increases slowly with temperature, and stabilizes at 63~65 ppm/°C in the high-temperature range. The α value range for epoxy resin TDE-85 is approximately 56~65 ppm/°C, close to E51, but slightly higher in the low-temperature range and slightly lower in the high-temperature range, with relatively small overall fluctuation. The α value range for bisphenol F epoxy resin is approximately 52~60 ppm/°C, significantly lower than the previous two, and remains the lowest throughout the entire temperature range, only rising to 59~60 ppm/°C in the high-temperature range. Therefore, bisphenol F epoxy resin has the smallest linear expansion coefficient, the best dimensional stability, and optimal thermal expansion performance, while E51 epoxy resin exhibits medium performance.

The main reason for the above phenomenon lies in the structural differences in the epoxy resins. Epoxy resin E51 (Bisphenol A type): The molecule contains a bisphenol A structure (two benzene rings connected by an isopropyl group). The isopropyl group provides a certain free volume, making segmental movement relatively easy. Therefore, its thermal expansion coefficient is moderate. TDE-85 (Cycloaliphatic epoxy resin): The molecule contains a cycloaliphatic structure with strong rigidity, but the conformation of the alicyclic ring might lead to larger distances between crosslinking points, and the crosslinking density is not necessarily high, resulting in a thermal expansion coefficient similar to E51. Bisphenol F epoxy resin: The two benzene rings in the molecule are connected by a methylene group (-CH_2_-), which is more compact than the isopropyl group in bisphenol A. The molecular chain is shorter, leading to higher crosslinking density after curing and more significant restriction of segmental movement. Therefore, its thermal expansion coefficient is the lowest [26].

The study of the coefficient of linear expansion of epoxy resin has been common in many fields. The aim of this study is to reduce the coefficient of linear expansion of the epoxy adhesive system by reducing the coefficient of linear expansion of the epoxy adhesive system under the premise of ensuring the toughness of the system, the bonding performance with the asphalt substrate, and the curing speed at room temperature, so that the coefficient of linear expansion of the epoxy adhesive system is more similar to the coefficient of linear expansion of the asphalt mixture compared with that of the other adhesive systems, and thus to reduce the risk of cracking of the anti-skidding system [27].

In addition, the above assertions are based on empirical and theoretical analyses, and further research is needed for specific cases. On the one hand, the temperature range should be expanded to −50 °C~100 °C, which is more suitable for the actual situation, but the results obtained in this research will gradually melt in this temperature range; therefore, further research is still needed. On the other hand, further research is needed on whether the linear expansion coefficient of the epoxy adhesive system has an effect on the crack resistance of the epoxy resin anti-slip system and to what extent.

(3) Cracking Performance

**Figure 19:**
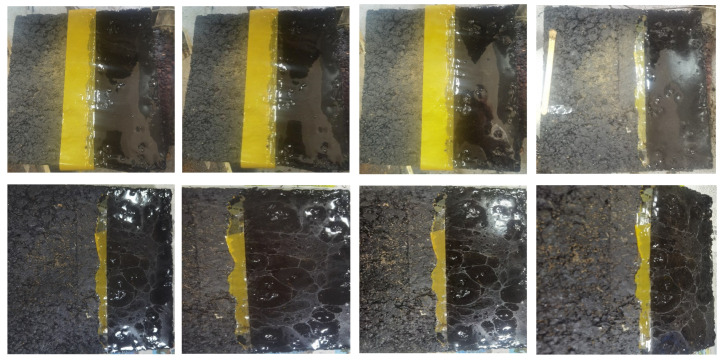
Effect of temperature on the cracking performance of epoxy adhesive systems.

To characterize the cracking performance of the epoxy system, tests were conducted according to Section 2.3 (10), and the specific results are shown in Figure 19.

As can be seen from Figure 19, the uncoated surface developed cracks and exhibited spalling, while the surface coated with the epoxy adhesive only showed cracks on the epoxy coating itself, with the underlying asphalt surface remaining intact and undamaged. The above test results demonstrate that the self-developed epoxy curing system proposed in this study can effectively alleviate surface cracking and spalling of rutting test specimens to a certain extent and thus improve the crack resistance of asphalt pavements under temperature-fluctuating conditions.

#### 3.5.3. Application

(1) Materials

**Figure 20:**
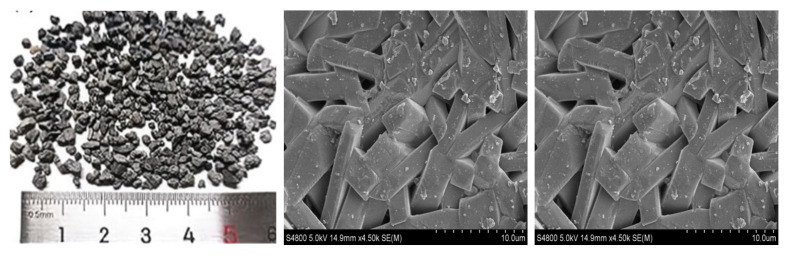
Macroscopic and microscopic photographs of bauxite clinker.

According to the results of the laboratory study, the amount of epoxy resin sprayed is about 2.1–2.2 L/m^2^, and the amount of bauxite (Figure 20) sprayed is about 11–12 kg/m^2^ [28]. The self-made curing agent is used as Component A, commercial liquid epoxy resin and other additives are used as Component B, and Components A and B are stirred evenly by electric stirring rod before paving.

The anti-slip aggregate is the crushed particles of 88 # bauxite with a particle size of 1–3 mm, the Al_2_O_3_ content is ≥88% (by quality), water absorption ≤3%, maximum size ≤4.75 mm, and the macro- and micro-morphologies are shown in Figure 20.

(2) Application

**Figure 21:**
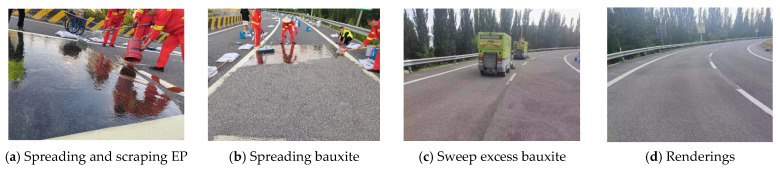
Photos of entity apps.

Based on the above research results, combined with practical engineering applications, the construction process and structure are shown in Figure 21.

After a certain period of time, the anti-slip surface structure began to form primary strength. The curing time is different according to the different temperature and length, generally 3–4 h in summer, and the road can be opened after complete curing. Finally, the anti-skid performance of the fully solidified pavement is tested, and the test results are as follows: the anti-skid pendulum value is 77, and the structural depth is 1.57 mm, which is much higher than the current code for pavement anti-skid performance requirements, indicating that the anti-skid surface has excellent anti-skid performance.

## 4. Conclusions

(1) In this study, a long-chain aliphatic amine (TETA) was employed as the base for the synthesis of epoxy resin Curing Agent B containing flexible chain segments and fast curing at room temperature by chain extension and Mannich reaction, with the optimal ratio of *n* (Cardanol):*n* (TETA):*n* (F):*n* (BDDE) = 1:1.4:0.8:0.7 (molar ratio of reactants). The optimal synthesis process was as follows: chain expansion reaction at 65 °C, reaction time of 3 h; Mannich reaction at 80 °C, reaction time of 120 min.
(2) BDDE can undergo a chain expansion reaction with TETA, embedding the flexible long-chain segments into the short-chain molecules of TETA, thereby effectively improving the toughness of the epoxy-curing system. When *n* (TETA):*n* (BDDE) = 2:1 (molar ratio of reactants), the elongation at break of the epoxy-curing system was 40.4%, the tensile strength was 7.1 Mpa, and the bond strength with the asphalt substrate was 6.8 Mpa.
(3) Epoxy-terminated polyurethane (EPU) prepolymer can improve the toughness of epoxy resin under the premise of not changing the mechanical properties of epoxy resin. When the amount of EPU prepolymer for the epoxy resin mass was 10%, its epoxy-curing system elongation at break reached 44.3%, tensile strength of 7.0 MPa, and asphalt substrate bonding strength of 6.9 Mpa were better than commercially available epoxy-curing systems.
(4) When *m* (EP):*m* (Curing agent B) = 1:1 (mass ratio), the epoxy system in the low-temperature (0 °C), room temperature (25 °C) and high-temperature (60 °C)conditions under hardening times of 240 min, 180 min and 20 min, respectively, was much lower than the commercially available epoxy-curing system. Particularly at low temperatures, the 35 min application period and 180 min maintenance time provide ample construction time for the project and, at the same time, ensure rapid post-construction maintenance, thus facilitating early traffic opening.
(5) The temperature shrinkage performance test results reveal that the temperature significantly influences the temperature shrinkage performance of the epoxy resin adhesive system, and its deformation is the largest in the high-temperature environment. Combined with the application scenarios, in the range of −30 °C to 100 °C, Bisphenol F epoxy resin showed the best thermal expansion performance, E51 showed moderate performance, and TDE-85 showed the worst.
(6) The epoxy resin adhesive system prepared by this research is characterized by high toughness, low shrinkage, strong adhesion with asphalt substrate, and fast curing at room temperature. Combined with the entity application, results show the epoxy resin anti-skid system prepared by the Institute, its fully cured pendulum value of 77, and structural depth of 1.57 mm, indicating a favorable application effect.

## Figures and Tables

**Table 1:** Technical specifications of epoxy resins.

Property	E51	TDE-85	Bisphenol F170
Appearance	Pale yellow, amber or colorless transparent liquid		
Viscosity (25 °C)/mPa·s	12,000~15,000	3000~6000	2000~5000
Epoxy Value/(eq/100 g)	0.48~0.54	0.80~1.00	0.55~0.62
Epoxy Equivalent/(g/eq)	184~190	100~125	160~180
Inorganic Chlorine/%	≤0.005	≤0.005	≤0.005
Volatile Content/%	≤0.5	≤1.0	≤0.3

**Table 2:** Effect of reaction temperature on amine value and viscosity of the system.

Reaction Temperature/°C	Amine Value/mg KOH·g^−1^	Viscosity/Pa·s
55	296.6	0.25
60	416.5	0.39
65	443.1	0.40

**Table 3:** Effect of reaction time on amine value and viscosity of the system.

Reaction Time/h	Amine Value/mg KOH·g^−1^	Viscosity/Pa·s
2	416.5	0.27
3	441.5	0.41
4	318.8	0.41

**Table 4:** Effect of reaction ratio on amine value and viscosity of the system.

TETA/BDDE (Molar Ratio of Reactants)	Amine Value/mg KOH·g^−1^	Viscosity/Pa·s
1/1	257.1	0.38
2/1	280.4	0.48
3/1	462.0	0.41

**Table 5:** FTIR peak assignments and analysis (Figure 7a).

Position/cm	Assignment	Variation and Explanation
1642	C-H bond	Present only in BDDE
1095	C-O bond	Present only in BDDE
1585	N-H bond	Present only in TETA
1594	C-H bond + N-H bond	Original N-H peak at 1585 cm^−1^ blue-shifted to 1594 cm^−1^
1126	C-O bond	Present in BDDE and chain-extended product; absent in TETA

**Table 6:** FTIR peak assignments and analysis (Figure 7b).

Position/cm	Assignment	Variation
3318	O-H bond + N-H bond	Peak shape of the broad N-H peak in TETA changed
1593	C=C bond	New peak
1456	C-N bond	New peak, stable wavelength
1132	C-N bond + C-O bond	New peak; original TETA peak (1126) and cardanol peak (1241) disappeared

**Table 7:** FTIR peak assignments and analysis (Figure 7c).

Position/cm	Assignment	Variation
3275	O-H bond + N-H bond	Introduces more phenolic hydroxyl and ether bonds than hardener A (3318)
1593	C=C bond	New peak
1465	C-N bond	New peak, stable wavelength
1132	C-O bond	Contains more ether bonds, and its peak position differs slightly from that of hardener A

**Table 8:** ^1^H NMR signal assignments for Curing Agent A.

Chemical Shift/ppm^−1^	Multiplicity	Integral	Assignment
6.98	t	1H	Cardanol benzene ring H-5
6.52	m	3H	Ditto, H-2, H-4, H-6 (overlapping).
5.43–5.19	m	4H	O-CH_2_- hydroxymethyl protons, from methylation of cardanol with formaldehyde.
5.05–4.81	m	1H	
2.87–2.52	m	37H	TEAT segment methylene (-CH_2_-NH-), -NH-CH_2_- methylene bridge from TETA and methylolated cardanol.
2.46–2.25	m	5H	Cardanol side chain Ar-CH_2_- and allylic -CH_2_-CH=CH-.
1.94	m	4H	Hydrogen on the terminal methyl group of the long alkyl chain.
1.48	q	3H	Specific methylene in alkyl chain.
1.44–1.10	m	16H	Long chain alkyl methylene -(CH_2_)_x_-.
0.81	m	3H	Terminal methyl -CH_3_.

Note: The integration values in the table are relative to H.

**Table 9:** ^13^C NMR key signal assignments for Curing Agent A.

Chemical Shift/ppm^−1^	Assignment
157.7	Cardanol C-OH (phenolic carbon).
143.6	Cardanol C-1 (connected to hydroxyl).
136.2–126.2	Benzene ring carbons and olefinic carbons (multiple signals, mixture).
118.3, 115.4, 114.2, 112.5	Benzene ring CH carbons.
60.3, 56.4, 54.0, 52.8, 51.8, 51.6, 48.6	60.3 ppm assigned to oxygen-connected methylene carbon, -O-CH_2_-, others, -NH-CH_2_- methylene bridge from TETA and methylated cardanol.
45.4–38.0	Methylene carbons in TETA segment and those adjacent to amines.
35.6–13.4	Methylene and terminal methyl protons in the cardanol long alkyl chain

**Table 10:** ^1^H NMR signal assignments for Curing Agent B.

Chemical Shift/ppm^−1^	Multiplicity	Integral	Assignment
7.05	t (J = 7.6 Hz)	1H	Cardanol benzene ring H-5 (ortho coupling).
6.70–6.48	m	3H	Cardanol benzene ring H-2, H-4, H-6.
5.35	m	4H	Hydroxymethyl protons -O-CH_2_- (Same as Table 5).
5.11–4.86	m	1H	
3.76–3.17	m	13H	Methylene protons of polyether chain -O-CH_2_-CH_2_-O-, likely from BDDE.
2.93–2.60	m	19H	-NH-CH_2_- (same as Table 5).
2.55–2.31	m	6H	Methylene adjacent to benzene ring in cardanol side chain Ar-CH_2_-, other aliphatic methylenes.
2.07–1.90	m	4H	Allylic methylene -CH_2_-CH=CH-.
1.68–1.46	m	4H	Internal methylenes of long alkyl chain.
1.29	m	16H	Long-chain alkyl methylene chain -(CH_2_)_x_-.
0.87	m	3H	Terminal methyl -CH_3_.

Note: The integration values in the table are relative to H.

**Table 12:** Adhesive strength of epoxy resin adhesive.

Epoxy Resin/Curing Agent	Tensile Strength/MPa	Elongation at Break/%
1/1	7.12	40.41
1/0.75	8.44	28.94
1/0.5	11.19	20.03
1/0.25	14.84	11.30

**Table 13:** Test results of the adhesive strength of the epoxy resin adhesive.

Epoxy Resin/Curing Agent (Mass Ratio)	Adhesive Strength on Asphalt Base/MPa	Adhesive Strength on Cement Base/MPa
1/1	6.83	8.07
1/0.75	4.58	7.19
1/0.5	4.27	5.96
1/0.25	3.32	4.57
Interface failure mode	Interlayer failure	Cement base failure

**Table 14:** Experimental results on the effect of EPU prepolymer on the properties of epoxy resin.

Content/%	Elongation at Break/%	Tensile Strength/MPa	Adhesion Strength/MPa	Impact Toughness/(J/cm^2^)
0	40.4	7.1	6.8	1.71
5	41.8	7.1	6.8	1.72
10	44.3	7.0	6.9	1.77
15	37.8	5.6	6.5	1.81
20	28.0	4.9	5.9	1.89

**Table 15:** Comparison of mechanical properties of different epoxy toughening–curing systems.

Type	Epoxy Resin/Curing Agent (Mass Ratio)	Bonding Strength/MPa		Elongation at Break/%	Tensile Strength/MPa
		Asphalt Base Surface	Cement Base Surface		
Self-made 1	1/1	4.5	6.8	28.7	11.4
Self-made 2	1/1	6.8	8.1	40.4	7.1
Self-made 3	1/1	6.9	8.3	44.3	7.0
TCI	1/1	5.9	7.7	31.2	6.4
D230	1/3	3.2	4.0	22.3	4.2
D400	3/5	2.8	3.5	47.3	0.7
D2000	5/2	0.8	1.0	82.1	0.4

**Table 16:** Low-temperature (0 °C) curing test results of different epoxy systems.

Type	Epoxy Resin/Curing Agent (Mass Ratio)	Pot Life	GEL Time	Curing Time
Self-made 1	1/1	100 min	170 min	235 min
Self-made 2	1/1	90 min	180 min	230 min
Self-made 3	1/1	90 min	180 min	240 min
Commercial TCI	1/1	80 min	120 min	160 min
D200	1/3	400 min	460 min	1 d
D400	3/5	510 min	590 min	3.5 d
D2000	5/2	28 h~32 h	14 d+	14 d+

**Table 17:** Normal-temperature (25 °C) curing test results of different epoxy systems.

Type	Epoxy Resin/Curing Agent (Mass Ratio)	Pot Life	Gel Time	Curing Time
Self-made 1	1/1	30 min	48 min	173 min
Self-made 2	1/1	36 min	45 min	175 min
Self-made 3	1/1	35 min	47 min	180 min
Commercial TCI	1/1	39 min	50 min	190 min
D230	1/3	252 min	240 min	1 d
D400	3/5	277 min	337 min	1.5 d
D2000	5/2	10 h~12 h	7 d+	7 d+

**Table 11:** ^13^C NMR key signal assignments for Curing Agent B.

Chemical Shift/ppm^−1^	Assignment
157.6	Phenolic carbon
144.4 (2C)	Aromatic carbon connected to alkyl chain in cardanol (C-1 or C-3)
136.7–112.9	Cardanol aromatic CH carbons and side chain olefinic carbons (multiple signals)
119.1, 115.8, 114.7, 112.9	Cardanol aromatic CH carbons
71.1, 68.6	Ether bond carbons -O-CH_2_- in polyether chain (from BDDE unit)
60.4	-CH-OH carbon from epoxy ring opening/Mannich base Ar-CH_2_-N carbon
54.3, 53.1, 51.5, 48.8, 48.6, 45.8	Mannich base Ar-CH_2_-N carbons and methylene carbons adjacent to amines -CH_2_-N<
41.2, 41.1, 38.3, 35.9	Other aliphatic methylene carbons (TEAT segments or linking units)
31.7–22.6	Dense multiple signals from methylene carbons of the cardanol alkyl chain and BDDE butanediol chain
14.1, 13.8	Cardanol terminal methyl -CH_3_ (different components)

**Table 18:** High-temperature (60 °C) curing test results of different epoxy systems.

Type	Epoxy Resin/Curing Agent (Mass Ratio)	Pot Life	Gel Time	Curing Time
Self-made 1	1/1	8 min	8 min	21 min
Self-made 2	1/1	7 min	7 min	20 min
Self-made 3	1/1	7 min	7 min	20 min
Commercial TCI	1/1	5 min	7 min	12 min
D230	1/3	25 min	40 min	1.8 d
D400	3/5	40 min	90 min	2 d
D2000	5/2	6 h~8 h	4 d	5 d~6 d

Note: In the table, “min” and “d” are time units representing minutes and days, respectively.

## Data Availability

The original contributions presented in this study are included in the article. Further inquiries can be directed to the corresponding author.

## Abbreviations

The following abbreviations are used in this manuscript:

BDDE	1,4-Butanediol diglycidyl ether
EP	Epoxy resin
EPU	Epoxy-terminated polyurethane
F	Formaldehyde
NBR	Nitrile butadiene rubber
PU	Polyurethane
TETA	Triethylenetetramine

## References

[1] Guo F.C., Pei J.Z., Li R., Zhou B.C., Chen Z.X. (2021). Study on the skidresistance of asphalt pavement: A state-of-the-art review and future prospective. Constr. Build. Mater..

[2] Mao S.X. (2021). The Common construction methods and bonding materials for colored anti slip thin layers on highways. Commun. Sci. Technol..

[3] Li X.T. (2020). The Research on epoxy anti-skid sealing technology for asphalt pavement. Sci. Technol. Ecnomy Mark..

[4] Xu H., Kong L., Zhang Y., He Z., Ran L. (2024). Preparation and characterization of polyurethane composite modified epoxy resin for novel colored Anti-skid pavement Materials. Mater. Lett..

[5] Sun P., Liang X., Ding Y., Wang T., Xiang S., Cai D. (2020). Laboratory evaluation on water-based and flexible epoxy/SiO_2_ nanocomposites to enhance anti-sliding effectiveness of pavement. Mater. Res. Express.

[6] Li Y., Yang X.Y., Lu B. (2023). Preparation and curing properties of waterborne epoxy emulsified asphalt/DMP-30 Composites. Case Stud. Constr. Mater..

[7] Lei J.A., Zhao F.J., Wang Y.Y., Ren X.F. (2024). Research on surface treatment technology for quickly improving the skid resistance of tunnel concrete Pavement. PLoS ONE.

[8] Guo X., Xie K., Liu Z., Elchalakani M.F., Zhou Y. (2023). A study on the bond strength of interface between Post-fire concrete and different epoxy resin Adhesives. Constr. Build. Mater..

[9] Sim K.-B., Lee T.-H., Han G.-Y., Kim M.-S. (2023). Thermal expansion and mechanical properties of urethane-modified epoxy bonded CFRP/steel joints at low and high temperatures for automotive. Compos. Struct..

[10] Wu Z.C., Chen Q.Y., Liu D.X., Fan J.S., Zhang Q.G., Chen W.J. (2023). In situ monitoring of epoxy resin curing process: Using glass transition as a Bridge [J]. Polym. Test..

[11] Lan L.Q., Yang K.R., Chen Y.L. (2024). Synthesis and curing reaction kinetics of bio-based phenalkamine curing agents. J. Minjiang Univ..

[12] (2024). Determination of Viscosity of Adhesives—Rotational Viscometer Method.

[13] (2021). Test Methods for Tensile, Compressive and Flexural Properties of Resin Castable Material.

[14] (2019). General Rules for Analysis with Infrared Spectroscopy.

[15] (2016). Plastics-Thermogravimetry (TG) of Polymers-Part 1: General Principles.

[16] Liu Y.X., Tai C.Q., Wang Y.L. (2014). Preparation of cashew phenol-modified amine epoxy resin curing agents. China Adhes. Y.

[17] Cui D.X. (2018). Effect of cashew phenol-modified polyethylene polyamines on the properties of epoxy resin curing compounds. Adhesion.

[18] Li S.H., Yang X.J., Li M. (2013). Structure of new cashew phenol-based unsaturated resins and their UV curing behaviour by infrared spectroscopic study. Spectrosc. Spectr. Anal..

[19] Pathak K.S., Rao S.B. (2006). Structural effect of phenalkamines on adhesive viscoelastic and thermal properties of epoxy networks. J. Appl. Polym. Sci..

[20] Song J. (2016). Preparation and Performance Research of Flexible Epoxy Resin. Ph.D. Thesis.

[21] Xie B.C. (2017). Preparation of Cashew Phenol Modified Phenalkamine Epoxy Curing Agent and Study on Adhesive Properties. Ph.D. Thesis.

[22] Wang Y.L. (2015). Synthesis of Cashew Phenol-Modified Amine Curing Agent and Its Application Study. Ph.D. Thesis.

[23] Tong W. (2018). Preparation and Properties of Long-Chain Glycidyl Esters and Their Modified Amines. Ph.D. Thesis.

[24] Zhang Y.Y., Wei J.J., Kong L., Li J.Q., Xu H. (2021). Preparation and characterization of PTMG-NDI type polyurethane modified epoxy resin. New Chem. Mater..

[25] Chen X.D., Shi J.B. (2025). Characterisation and analysis of low-temperature linear expansion coefficients of epoxy resins with different molecular structures. Energy Conserv. Technol..

[26] Yi R.J., He J.X., Sun T. (2024). Preparation and performance evaluation of low linear expansion coefficient thermally conductive epoxy resin. China Plast. Ind..

[27] Li Z. (2022). Research on Low-Temperature Thermal Expansion Properties of Epoxy Resin and Its Composite Materials. Ph.D. Thesis.

[28] Li Y.F., Nong B.Y. (2019). Highway epoxy resin system anti-skid surface layer construction technology application research. West. China Commun. Sci..

